# First transcriptome analysis of the winter tick (*Dermacentor albipictus*) reveals sex-specific expression patterns and potential targets for genetic control

**DOI:** 10.1093/g3journal/jkaf116

**Published:** 2025-05-23

**Authors:** Rhodri T M Edwards, Igor Antoshechkin, Eddie Hill, Michael W Perry, Pia U Olafson, Perot Saelao, Kimberly H Lohmeyer, Omar S Akbari

**Affiliations:** School of Biological Sciences, Department of Cell and Developmental Biology, University of California San Diego, La Jolla, CA 92093, USA; Division of Biology and Biological Engineering (BBE), California Institute of Technology, Pasadena CA91125, USA; School of Biological Sciences, Department of Cell and Developmental Biology, University of California San Diego, La Jolla, CA 92093, USA; School of Biological Sciences, Department of Cell and Developmental Biology, University of California San Diego, La Jolla, CA 92093, USA; Livestock Arthropod Pests Research Unit, USDA-ARS, Kerrville, TX 78028, USA; Veterinary Pest Genetics Research Unit, USDA-ARS, Kerrville, TX 78028, USA; Livestock Arthropod Pests Research Unit, USDA-ARS, Kerrville, TX 78028, USA; School of Biological Sciences, Department of Cell and Developmental Biology, University of California San Diego, La Jolla, CA 92093, USA

**Keywords:** RNA-Seq, transcriptome, winter tick, *D. albipictus*, vector control, pgSIT, *doublesex*

## Abstract

The winter tick, *Dermacentor albipictus*, is a significant North American ectoparasite, posing health risks to ruminants and occasionally humans. Despite its ecological importance, limited genomic resources exist for this species. This study provides the first comprehensive transcriptomic analysis of the recently published winter tick genome, focusing on tissues isolated from early-stage embryos, sexed adults, dissected ovaries, and dissected male reproductive systems. We identified the most abundant gene ontologies and analyzed differential gene expression. Differential gene expression revealed significant sex-biased expression patterns, and functional annotations identified candidate genes involved in sex determination. Notably, we identified the first documented case of sex-specific splicing of a doublesex-like gene in chelicerates, a mechanism previously thought to be absent in this clade. These transcriptome data serve as a critical resource for understanding the biology of *D. albipictus* and will facilitate the development of novel genetic technologies aimed at population suppression and reduction of tick-borne illnesses.

## Introduction

Winter ticks, *Dermacentor albipictus* (phylum: Arthropoda; subphylum: Chelicerata; class: Arachnida; order: Ixodida; family: Ixodidae), are hard tick ectoparasites that predominantly infest ruminants—such as moose (Samuel 1989; Calvente *et al*. 2020), elk, and white-tailed deer (Calvente *et al*. 2020; Machtinger *et al*. 2021) across North America (Chenery *et al*. 2023). They also parasitize domestic bovids and equines (Sundstrom *et al*. 2021) and domestic cats and dogs (Duncan *et al*. 2020).

Winter tick males and females both bloodfeed. Females require larger blood meals to support egg production, resulting in significant engorgement, while males feed intermittently and ingest smaller volumes (Kutz *et al*. 2012). Infestations can reach tens of thousands of ticks per host, leading to substantial blood loss, hair loss, and host stress, which can negatively impact survival, particularly in calves (McLaughlin and Addison 1986; Duncan *et al*. 2020; Rosenblatt *et al*. 2021). Like other members of Ixodidae (e.g. *Ixodes scapularis*), winter ticks may also serve as vectors for atypical bacterial pathogens when blood feeding. Studies have detected *Anaplasma* spp. (Ewing *et al*. 1997; Baldridge *et al*. 2009), *Francisella*-like endosymbionts (Baldridge *et al*. 2009), and *Borrelia burgdorferi* (Magnarelli *et al*. 1986; Kocan *et al*. 1992) in winter ticks. These pathogens are respectively associated with diseases such as anaplasmosis, tularemia, and Lyme disease, which can result in a host of mild to severe clinical symptoms including fever, myalgia, and glandular swelling. These diseases can be fatal if left untreated.

While winter ticks primarily parasitize nonhuman mammals, they are not without risk to humans. A review of hard tick bites in humans showed that there have been 465 (0.2%) bites from *D. albipictus* in the United States from 1910 to 2017 compared to 158,008 (67.3%) from *I. scapulari*s in the United States from 1909 to 2021 (Eisen 2022). Winter ticks largely affect hunters exposed to moose and deer (Rand *et al*. 2007). In humans, *D. albipictus* has been implicated in the transmission of *Babesia duncani*, a potentially fatal pathogen that can cause babesiosis (Swei *et al*. 2019).

Tick-borne zoonotic diseases are second only to mosquito-borne diseases in incidence and variety of pathogens (de la Fuente *et al*. 2008). Alarmingly, tick–host interactions are expected to increase in number in response to global climate change (Swei *et al*. 2019; Gilbert 2021; Pouchet *et al*. 2024). Current control strategies primarily focus on personal protection, landscape and vegetation management, and acaricide use (Eisen 2021). These are not without risk, however, as the indiscriminate use of acaricides is partly responsible for the increase in reports of multiresistant tick strains (Agwunobi, Yu, and Liu 2021). There is a need to develop new methods for control, including vaccine targets and genetic tools.

The development of genetic tools to reduce ticks and tick-borne illnesses requires a thorough understanding of tick phylogeny, genetics, and transcriptomics. Currently, 16 complete reference genomes are available for Ixodidae tick species via NCBI (Jia *et al*. 2020; Guerrero *et al*. 2021; Chou *et al*. 2023; De *et al*. 2023). Tissue-specific transcriptome studies in ticks have analyzed expression in dissected tissues that have significance to feeding and reproduction: midgut (Xu *et al*. 2016; Omondi *et al*. 2023), salivary glands (Schwarz *et al*. 2013; Giachetto *et al*. 2019; Pienaar *et al*. 2021), male reproductive system (Sonenshine *et al*. 2011), and ovaries (Zhao, Qu, and Jiao 2021; Cossío-Bayúgar *et al*. 2024). However, time points and tissues that would be most useful for genetic tool development are lacking.

In light of the recently published chromosome-level assembly of the *D. albipictus* genome (GCA_038994185.2), the goal of this study was to advance our understanding of the genetic structure of *D. albipictus* (and consequently, Ixodidae and Chelicerata in general) and to identify potential targets that can be used to create genetic tools geared toward population suppression of *D. albipictus.* To do this, we analyzed the transcriptomes of *D. albipictus* in early-stage embryos (when genetic constructs are typically introduced for transgenesis), sexed adults, dissected ovaries, and dissected male reproductive systems. We identified the most abundant gene ontologies and analyzed differential gene expression. Sex-specific expression patterns helped us identify a putative *doublesex* gene that could potentially be leveraged in precision-guided sterile insect technique (pgSIT) to create sterile or intersex males and subsequently reduce the *D. albipictus* population.

## Methods

### Tick rearing

The *D. albipictus* colony (Rhodes 1998 strain) used in this study was propagated on cattle at the Knipling-Bushland US Livestock Insects Research Laboratory (KBUSLIRL; Kerrville, TX, United States). Larval and engorged female *D. albipictus* are incubated in glass aquaria with temperature and humidity maintained at 24°C, 92–97% RH. Humidity was regulated with a potassium nitrate supersaturated salt solution at the base of the aquarium. A bovine calf was infested with *D. albipictus* larvae under a muslin cloth patch, which was adhered to the shaved region of the animal using a veterinary-approved adhesive. Females that fed to repletion and dropped from the host were collected, and ovaries from pre-oviposition females were dissected 10 days postdrop. For collection of egg masses, engorged females (*n* = 20) were placed dorsal side down on double-sided sticky tape adhered to the interior of a Petri dish to await oviposition. At the onset of oviposition, eggs deposited over a 6-h period were collected, snap frozen in liquid nitrogen, macerated in Monarch DNA/RNA Protection Reagent (NEB), and stored at −80°C. Unfed adult females and males were obtained from the host at 18–19 days after larval infestation, which coincides with the nymphal molt to adults. For the collection of reproductive systems from fed, unmated males, newly molted adults were obtained from the host and transferred to a separate, male-only patch on the same host. The males attached and were allowed to feed for 4 days, after which they were pulled from the host for dissection. All dissected tissues were transferred to Monarch DNA/RNA Protection Reagent, macerated, and stored at −80°C before extraction. Three biological replicates of each sample type were analyzed, yielding a total of 15 samples.

### RNA extraction

Illumina RNA sequencing was conducted to quantify gene expression patterns. RNA was extracted from all life stages and tissues using the Monarch Total RNA Miniprep Kit (NEB), following the manufacturer's protocol.

After RNA extraction, DNase treatment was conducted using RNase-free DNase I. The integrity of the RNA was assessed, and mRNA was isolated from the total RNA. RNA-Seq libraries were prepared for Illumina sequencing using the NEB library prep kit. Library sequencing was conducted using Illumina NextSeq 2000 to give paired end reads of 150 nt and 20 million reads per library. RTA1.18.64 was used for base calls and converted to FASTQ using bclfastq 1.8.4.

### Transcriptome assembly and annotation

RNA sequencing reads were trimmed to remove adapter sequences with AdapterRemoval (Schubert, Lindgreen, and Orlando 2016) and aligned to the GCA_038994185.2 genome with STAR (Dobin *et al*. 2013) using the 2-pass method. The 2-pass method enables more accurate quantification of spliced reads in the absence of annotations. The aligned reads for each sample were processed separately with StringTie (Pertea *et al*. 2015) to generate de novo transcript annotations, which were subsequently merged, with StringTie, to generate the final GTF file Dalb_finalv2.merged.trimmed_reads.gtf.

Transcript coordinates defined in Dalb_finalv2.merged.trimmed_reads.gtf were used to generate transcript sequences. Encoded ORFs were predicted using ORFfinder (Sayers *et al*. 2012), and the longest one for each transcript was parsed. Protein domains were identified with HMMSCAN (Eddy 2011), and Gene Ontology (GO) terms associated with PFAM domains were added using pfam2GO (Mitchell *et al*. 2015) mappings. Protein and transcript homologs in 2 tick species were identified by blast searches against proteomes and transcriptomes of *Dermacentor andersoni* (GCF_023375885.1) and *Dermacentor silvarum* (GCF_013339745.2).

### Gene quantification, differential expression, and GO enrichment analyses

Genes defined in Dalb_finalv2.merged.trimmed_reads.gtf were quantified with featureCounts (Liao, Smyth, and Shi 2014), and the count values were then transformed into transcripts per million (TPM) and fragments per kilobase of transcript per million mapped reads (FPKM) values with a Perl script. TPM values were used to perform principal component analysis (PCA) and clustering analyses. Plots were generated in R using ggdendro and ggplot2 packages (Kassambara 2015).

Differential analysis runs for 5 pairwise comparisons (embryo vs adult; male vs female; Mrs vs male; ovary vs female; Mrs vs ovary) were conducted with DESeq2 (Love, Huber, and Anders 2014). The identified differentially expressed genes were used to perform GO enrichment analyses for the biological process (BP) ontology terms using the topGO (Alexa and Rahnenfuhrer 2024) R package. Up- and downregulated gene sets were analyzed separately using the weight01 algorithm and Fisher's exact test.

### Sex-specific splicing and doublesex candidate identification

Differential exon usage analyses were performed with DEXSeq (Anders *et al*. 2012). Briefly, a flattened GFF file was generated with dexseq_prepare_annotation.py and exon parts were quantified with dexseq_count.py scripts provided by the DEXSeq package. Exon plots were generated with the plotDEXSeq function. *Doublesex* candidates were identified by parsing blast results that showed homology to genes annotated as *dsx* or *dsx-like* in *D. andersoni* and *D. silvarum*. Splice junction counts were extracted from SJ.out.tab files generated by the STAR aligner. Transcript structures and splicing patterns were visualized in Integrative Genomics Viewer (IGV) (Robinson *et al*. 2011).

## Results

### Overview of sequencing, gene expression, and PCA

Transcriptome sequencing was performed to provide an overview of gene expression in *D. albipictus* unfed males (ufMale), unfed females (ufFem), the male reproductive system (Mrs), ovaries preoviposition (Ov-preov), and 0–6 h old embryos (Embr_0-6h). Unfed adult samples were collected to provide comparisons between males and the pre-vitellogenic phase females (i.e. before oogenesis has occurred). Mrs and Ov-preov samples were collected to investigate differences in germline-specific gene expression in males and females. We generated an extensive dataset comprising 1.2 billion paired end 150 base reads with an average of 79.95 million reads per sample and a total yield of 332.8 Gb. Of the reads, 92.85% were mapped to the *D. albipictus* genome with 85.95% being uniquely mapped (Supplementary Tables 1 and 2).

**Fig. 1.**
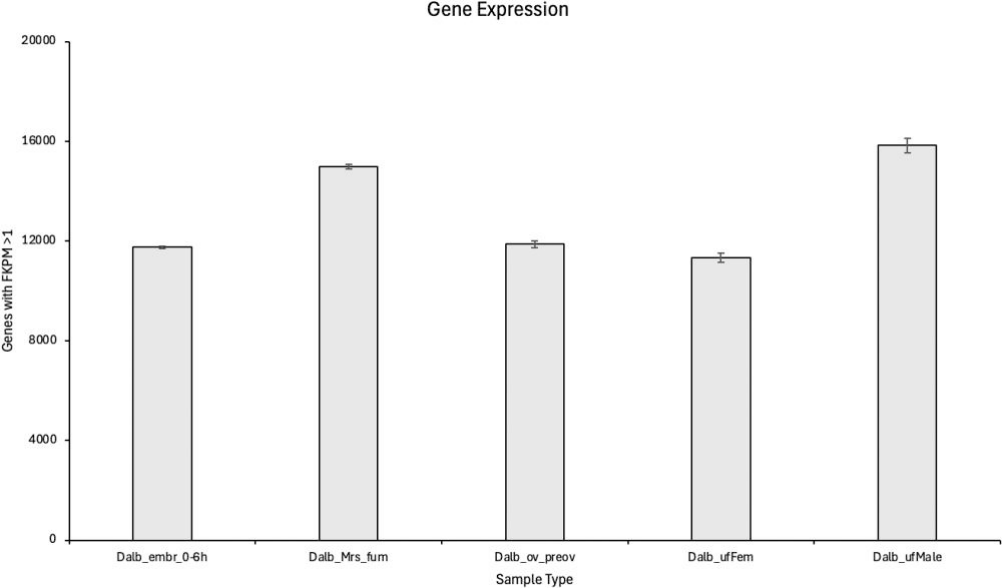
Number of expressed genes (FKPM > 1) for all *D. albipictus* sample types. Sample standard deviation (calculated with Bessel's correction, n-1) is shown for each group, illustrating the variability in gene counts across replicates of each sample.

**Fig. 2.**
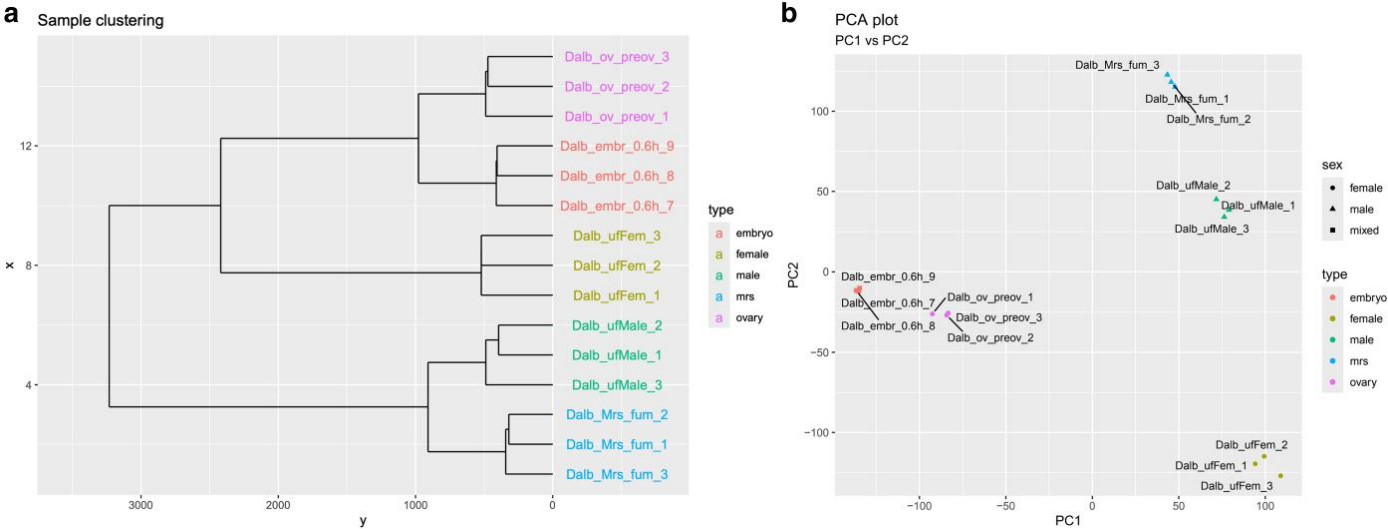
Clustering of *D. albipictus* RNA-Seq samples comparing male, female, and embryo sources of RNA. TPM values were used for clustering and PCA analyses. Dendrogram a) and PCA analysis b) showed the clustering of samples from the different sources of RNA.

Because no annotations were available for the GCA_038994185.2 genome, transcripts were assembled with StringTie for each of the sequenced samples individually and merged to generate a unified set of gene models comprising 22,680 genes and 47,811 transcripts. To provide a general overview of gene expression levels, the mean number of detected genes at FPKM > 1 for each sample type was determined and ranged from 11,336 in ufFem to 15,834 in ufMale. Embr_0-6h, Ov-preov, and Mrs samples expressed intermediate numbers of genes of 11,763, 11,882, and 14,986, respectively (Fig. 1; Supplementary Tables 1 and 3). Hierarchical clustering and PCA on TPM values were used to visualize relationships between samples and showed tight grouping of replicates with well-separated sample types, as expected (Fig. 2).

### Identification of prevalent functional annotations and gene ontologies

**Fig. 3.**
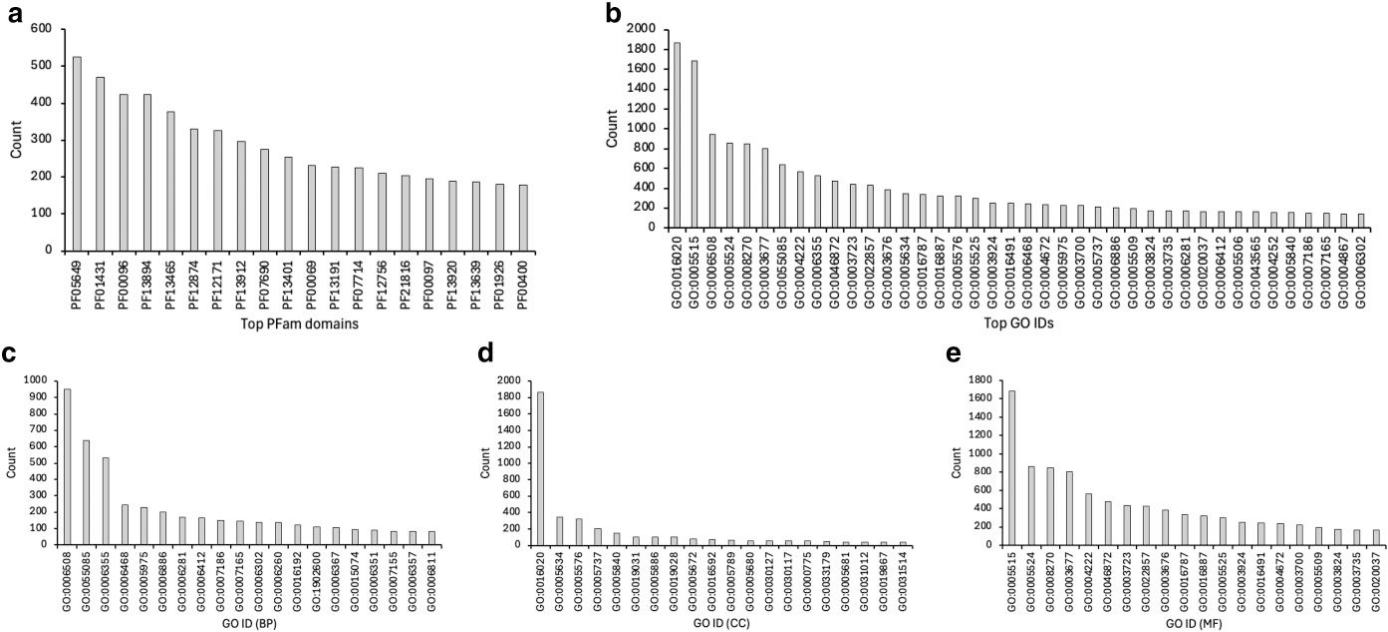
Identification of the most abundant Pfam domains and GO IDs assigned to sequences from *D. albipictus*. Transcripts were assembled and PFAM domain annotation was conducted using HMMSCAN a). GO terms were assigned to genes based on PFAM-to-GO mapping. Top 40 most frequent terms are shown b). The top 20 terms for each ontology (BP, MF, CC) were identified c, d, and e), respectively.

Once transcripts were assembled, functional annotation was conducted using ORFfinder and HMMSCAN. A total of 11,933 Pfam domains were identified; 6,870 were identified 2 or more times and 5,063 were identified once within the data. Approximately 70% (15,377 of 22,680) of the genes had at least 1 Pfam domain, with 4,382 genes annotated with a single Pfam domain and 10,995 genes annotated with multiple Pfam domains. The top 20 most abundant Pfam domains consisted of 11 linked to zinc finger domains (PF00096, PF13894, PF13465, PF12874, PF12171, PF13912, PF12756, PF21816, PF00097, PF13920, PF13639), 2 linked to M13 peptidase (PF05649 and PF01431), 2 AAA + ATPase domains (PF13401 and PF13191), a Protein kinase domain (PF00069), a Protein tyrosine and serine/threonine kinase (PF07714), a 50S ribosome-binding GTPase (PF01926), a major facilitator superfamily (PF07690), and a WD domain (PF00400) (Fig. 3a; Supplementary Table 4).

Genes (*n* = 10,861) were annotated with GO terms using PFAM-to-GO mapping with 7,733 genes associated with multiple terms. A total of 2,010 unique GO terms were assigned to the sequence data across all samples. The most numerous GO annotations were identified for sequences containing 1 or more annotations (Fig. 3, b–e; Supplementary Table 5). These included GO:0016020 (membrane), GO:0005515 (protein binding), GO:0006508 (proteolysis), GO:0008270 (zinc ion binding), GO:0005524 (ATP binding), GO:0003677 (DNA binding), GO:0055085 (transmembrane transport), GO:0004222 (metalloendopeptidase activity), GO:0006355 (regulation of DNA-templated transcription), and GO:0046872 (metal ion binding).

To provide additional insight into gene function, close homologs for predicted *D. albipictus* transcripts and proteins were identified in 2 annotated ticks from the *Dermacentor* genus, *D. andersoni* and *D. silvarum*. Transcripts encoded by 19,848 (87.5%) and 19,101 (84.2%) *D. albipictus* genes had significant blast hits (e-value < 0.01) against transcriptomes of *D. andersoni* and *D. silvarum*, respectively. A total of 16,624 (73.3%) and 16,153 (71.2%) genes displayed significant homology with *D. andersoni* and *D. silvarum* on the protein level (Supplementary Table 6).

### Differential gene expression and GO enrichment analyses

**Fig. 4.**
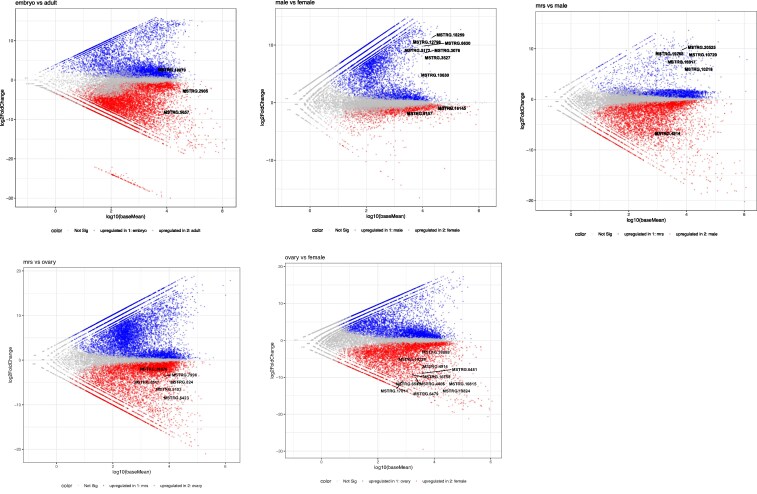
MA plots for pairwise analysis of differential gene expression in *D. albipictus* samples. Colored dots represent genes that are differentially expressed showing *P*-adjusted values < 0.05. Gray dots represent genes that are not differentially expressed. Genes highlighted in the plots indicate highly overexpressed genes in the comparative analyses (data presented in Supplementary Tables 13–22).

Five pairwise comparisons were performed to identify differentially expressed genes between Embr_0-6h and adults (Supplementary Table 7), ufMale and ufFem (Supplementary Table 8), Mrs and ufMale (Supplementary Table 9), Mrs and Ov-preov (Supplementary Table 10), and Ov-preov and ufFem (Supplementary Table 11; Fig. 4). Expression levels of 17,155, 12,171, 12,788, 17,622, and 14,360 genes were significantly affected (*P*adj < 0.05) between the 5 pairs of samples, respectively (Supplementary Table 12). Up- and downregulated gene sets identified in the comparisons were used to perform BP GO enrichment analyses to present a high-level overview of the observed changes.

For the Embr_0-6h vs adult pair, top BP GO terms enriched in genes upregulated in embryos included GO:0006281 (DNA repair), GO:0006355 (regulation of DNA-templated transcription), GO:0006260 (DNA replication), GO:0000398 (mRNA splicing), and GO:0000278 (mitotic cell cycle) (Supplementary Table 13). Top terms enriched in adults were GO:0006508 (proteolysis), GO:0005975 (carbohydrate metabolic process), GO:0030682 (symbiont-mediated perturbation of host defenses), GO:0006412 (translation), and GO:1900137 (negative regulation of chemokine activity) (Supplementary Table 13). Consistent with this, some of the genes most strongly upregulated in embryos include MSTRG.18879, a putative homolog of BRISC and BRCA1-A complex member 1-like protein, known to be involved in DNA repair-dependent chromatin remodeling and mitotic DNA damage checkpoint signaling. A number of genes encoding zinc finger containing proteins likely involved in various nucleic acid metabolic processes are also at the top of the embryonically enriched genes. On the other hand, a homolog of eukaryotic initiation factor 4A-I-like protein, MSTRG.2905, that is a part of the translation initiation complex, and MSTRG.5657, which encodes a lysosomal alpha-mannosidase-like protein involved in glycoprotein break down, as well as a number of genes encoding various cuticle proteins are upregulated in adults (Supplementary Table 14).

In ufMale vs ufFem comparison, GO:0006508 (proteolysis), GO:0005975 (carbohydrate metabolic process), GO:0030261 (chromosome condensation), GO:2001256 (regulation of store-operated calcium entry), and GO:0006614 (SRP-dependent cotranslational protein targeting to membrane) terms were strongly enriched in genes upregulated in males (Supplementary Table 15). In agreement with that, a number of peptidase-encoding genes, such as MSTRG.18259, MSTRG.10630, MSTRG.6630, and MSTRG.3076 were among top male-biased genes, as were genes involved in carbohydrate metabolism including MSTRG.3527, MSTRG.12798, and MSTRG.3172 (encodes a chitinase-3-like protein 1). Relatively few genes were upregulated in females and many of them did not have identifiable GO-annotated PFAM domains resulting in poor GO term enrichment with only GO:0006412 (translation) term being strongly significantly enriched. However, several genes with homologs identified in *D. andersoni* and *D. silvarum* are significantly upregulated in females, including MSTRG.9157, which encodes an ETS homologous factor and transcription factor MafA-like gene MSTRG.16145 (Supplementary Table 16).

The GO terms GO:0006508 (proteolysis), GO:0006302 (double-strand break repair), GO:0005975 (carbohydrate metabolic process), GO:0030261 (chromosome condensation), and GO:0007059 (chromosome segregation) were enriched in genes upregulated in Mrs compared to ufMale (Supplementary Table 17). Peptidases such as MSTRG.10729, MSTRG.10218, MSTRG.20525, MSTRG.19268, and MSTRG.16317 were among the strongest upregulated genes identified in this comparison (Supplementary Table 18). GO:0030682 (symbiont-mediated perturbation of host defenses) was the most enriched GO term in genes overexpressed in ufMale. Multiple proteins containing a histamine-binding domain, including MSTRG.4914 (a homolog of male-specific histamine-binding salivary protein), were strongly expressed in ufMale samples. Other top ufMale enriched GO terms included GO:0006694 (steroid biosynthetic process), GO:0006811 (monoatomic ion transport), GO:0006811 (monoatomic ion transport), and GO:1900137 (negative regulation of chemokine activity) (Supplementary Table 17).

Like in Mrs vs ufMale, Mrs vs Ov-preov comparison showed the enrichment of similar set of GO terms in Mrs samples, including GO:0006508 (proteolysis), GO:0005975 (carbohydrate metabolic process), and GO:0030261 (chromosome condensation) (Supplementary Table 19). Genes expressed at higher levels in ovaries were enriched in GO:0015074 (DNA integration), GO:0006355 (regulation of DNA-templated transcription), GO:0006281 (DNA repair), GO:0006886 (intracellular protein transport), and GO:0006801 (superoxide metabolic process). Genes annotated with these terms that were expressed at significantly higher levels in Ov-preov included ras-related protein Rab-32-like MSTRG.8423, forkhead box protein F1-like MSTRG.2103, coiled-coil domain-containing protein 22 homolog MSTRG.7996, K02A2.6-like MSTRG.5917, protein bicaudal C homolog 1-like MSTRG.824, and zinc finger protein 425-like MSTRG.20876 (Supplementary Table 20).

The ovary-enriched set in the Ov-preov vs ufFem comparison resembled the one above and includes such terms as GO:0015074 (DNA integration), GO:0006886 (intracellular protein transport), GO:0006260 (DNA replication), GO:0016192 (vesicle-mediated transport), and GO:1902600 (proton transmembrane transport) (Supplementary Table 21). Like in ufMale, GO:0030682 (symbiont-mediated perturbation of host defenses) was strongly enriched in ufFem. Many histamine-binding domain encoding genes that are likely involved in the suppression of the host immune response during the blood meal (Paesen *et al*. 1999) were overexpressed in females (MSTRG.19815, MSTRG.19824, MSTRG.4914, MSTRG.4465). Twelve out of 15 genes annotated with GO:1900137 (negative regulation of chemokine activity) in the genome were overexpressed in females compared to ovaries (e.g. MSTRG.6585, MSTRG.17014, MSTRG.6479, and MSTRG.6481). Genes encoding Inhibitor_I68 domain containing proteins and annotated with GO:0007596 (blood coagulation) were also strongly overexpressed in females. Many genes encoding structural components of ribosome and other translation related genes were also overexpressed: MSTRG.16750, MSTRG.18888, and MSTRG.19229 (Supplementary Tables 21 and 22).

### Identification of *doublesex* as a target for genetic control tools

In many insect species, the *doublesex* gene acts as a major sex determination factor. It has been used for engineering of genetic control tools (Kyrou *et al*. 2018; Kandul *et al*. 2019; Li *et al*. 2024). Our blast searches identified 4 genes that encode proteins homologous to doublesex-like proteins in *D. andersoni* and *D. silvarum:* MSTRG.10335, MSTRG.10337, MSTRG.18500, and MSTRG.9862.

MSTRG.10335 and MSTRG.10337 were located adjacent to each other on scaffold CM090517.1. MSTRG.10335 was robustly expressed in both male and female samples with slightly higher levels in males. However, it displayed a profound difference in splicing pattern between ufMale and ufFem as well as between Mrs and Ov-preov. Splice junction SJ004 appeared to be female-specific, while SJ005 and SJ006 appeared to be male-specific (Table 1). SJ004 belonged to MSTRG.10335.3 transcript, which was exclusive to ufFem and Ov-preov samples. SJ005 originated from MSTRG.10335.2, which was not expressed in Ov-preov and was expressed at very low levels in ufFem samples. SJ006 belonged to the MSTRG.10335.1 transcript, which was specific to ufMale and Mrs. In contrast to MSTRG.10335, MSTRG.10337 was identified as one of the strongly differentially expressed genes in the male vs female comparison: it was practically absent in female samples but was expressed at TPM of ∼9 in males (Table 2).

**Table 1.** Splice junction analysis of MSTRG 10335 showing the sex specificity of SJ004 (female) and SJ005 and SJ006 (male) and showing which transcripts these splice junctions belong to. Values represent splice junction counts for all sample replicates.

Junction ID	Belongs to transcripts	Mrs fum	Mrs fum	Mrs fum	Ov preov	Ov preov	Ov preov	ufFem	ufFem	ufFem	ufMale	ufMale	ufMale	Mrs mean	Ov mean	ufFem mean	ufMale mean
		1	2	3	1	2	3	1	2	3	1	2	3				
MSTRG.10335:SJ001	MSTRG.10335.1; MSTRG.10335.2	19	18	15	4	5	10	5	7	0	18	22	12	17.33	12.00	4.00	17.33
MSTRG.10335:SJ002	MSTRG.10335.1; MSTRG.10335.2	23	24	17	2	5	10	3	0	1	13	25	27	21.33	8.00	1.33	21.67
MSTRG.10335:SJ003	MSTRG.10335.1; MSTRG.10335.2	20	22	9	1	3	3	2	6	2	19	23	19	17.00	6.67	3.33	20.33
MSTRG.10335:SJ004	MSTRG.10335.3	0	0	0	3	2	8	1	1	1	0	0	0	0.00	4.33	1.00	0.00
MSTRG.10335:SJ005	MSTRG.10335.2	5	12	6	0	0	0	1	1	0	5	14	7	7.67	0.00	0.67	8.67
MSTRG.10335:SJ006	MSTRG.10335.1	8	6	2	0	0	0	0	0	0	15	7	18	5.33	0.00	0.00	13.33

**Table 2.** Expression of 4 putative doublesex genes identified in *D. albipictus* sequencing data showing count, TPM, and FPKM values for all samples including 0–6 h old embryos (Embr_0-6h), the male reproductive system (Mrs), ovaries preoviposition (Ov-preov), unfed females (ufFem), and unfed males (ufMale).

	Gene ID	Embr 0-6h_7	Embr 0-6h_8	Embr 0-6h_9	Mrs_fum1	Mrs_fum2	Mrs_fum3	Ov preov 1	Ov preov 2	Ov preov 3	ufFem 1	ufFem 2	ufFem 3	ufMale 1	ufMale 2	ufMale 3
Count	MSTRG.10335	74	121	133.5	1737.5	1789.5	1560.5	692.5	1018.5	787.5	2257.5	2160.5	1334.5	2624.25	3243.5	3377.5
	MSTRG.10337	711	885	816	333	294	254.25	49	73	90	16	28	23	996.5	1287.5	869.5
	MSTRG.18500	557.44	608.33	615.83	112.67	124.17	117	326.17	211.17	234	116	99	146.17	90.67	95.33	74
	MSTRG.9862	2879.25	3315	3919.5	695	563	402	1412	1707	2378	3930.5	2882	2954	4008	4300	4411
TPM	MSTRG.10335	0.22	0.32	0.34	3.76	3.62	3.60	2.14	2.57	1.94	5.07	5.24	3.14	5.98	7.31	8.54
	MSTRG.10337	7.93	8.72	7.61	2.66	2.20	2.17	0.56	0.68	0.82	0.13	0.25	0.20	8.39	10.73	8.12
	MSTRG.18500	5.50	5.30	5.08	0.80	0.82	0.88	3.30	1.74	1.89	0.85	0.78	1.12	0.68	0.70	0.61
	MSTRG.9862	11.62	11.83	13.23	2.01	1.52	1.24	5.84	5.77	7.85	11.80	9.34	9.29	12.22	12.97	14.91
FPKM	MSTRG.10335	0.08	0.12	0.12	1.71	1.61	1.57	0.81	1.00	0.78	2.26	2.39	1.39	2.71	3.21	3.77
	MSTRG.10337	2.98	3.18	2.82	1.21	0.98	0.94	0.21	0.27	0.33	0.06	0.11	0.09	3.80	4.70	3.58
	MSTRG.18500	2.06	1.93	1.88	0.36	0.37	0.38	1.24	0.68	0.76	0.38	0.36	0.50	0.31	0.31	0.27
	MSTRG.9862	4.36	4.32	4.90	0.91	0.68	0.54	2.20	2.25	3.15	5.25	4.27	4.12	5.53	5.69	6.58

MSTRG.9862 was also on the CM090517.1 scaffold, more than 50 Mb away from the MSTGR.10335/MSTGR.10337 locus. MSTRG.9862 was expressed strongly at ∼10–12 TPM levels in both males and females and displayed no differential splicing. MSTRG.18500 was located on scaffold CM090522.1 and showed no differential expression or splicing between male and female samples. It was also expressed at low levels in all samples except for Embr_0-6h.

### Analysis of global sex-specific splicing patterns

To identify potential additional targets for splicing-based strain engineering, we performed exon utilization analysis between male and female samples on the whole genome level. Among 19,219 genes with multiple transcripts, 6,852 showed evidence of sex-specific splicing (*P*adj < 0.1), affecting 26,814 out of 190,156 exons (Supplementary Table 23). Exon utilization analysis identified differentially expressed exons while controlling for differences due to overall gene expression levels in the samples. However, the analysis could not fully reconstruct alternatively spliced transcript structures and was highly sensitive to the quality of the genomic annotations. Therefore, manual examination of transcript structures and read alignments was necessary to confirm the validity of identified hits. While the examination of the top candidates generated by the analysis identified several false positives due to incorrect gene annotations (e.g. chimeric transcripts), multiple true sex-specific splicing events were identified.

**Fig. 5.**
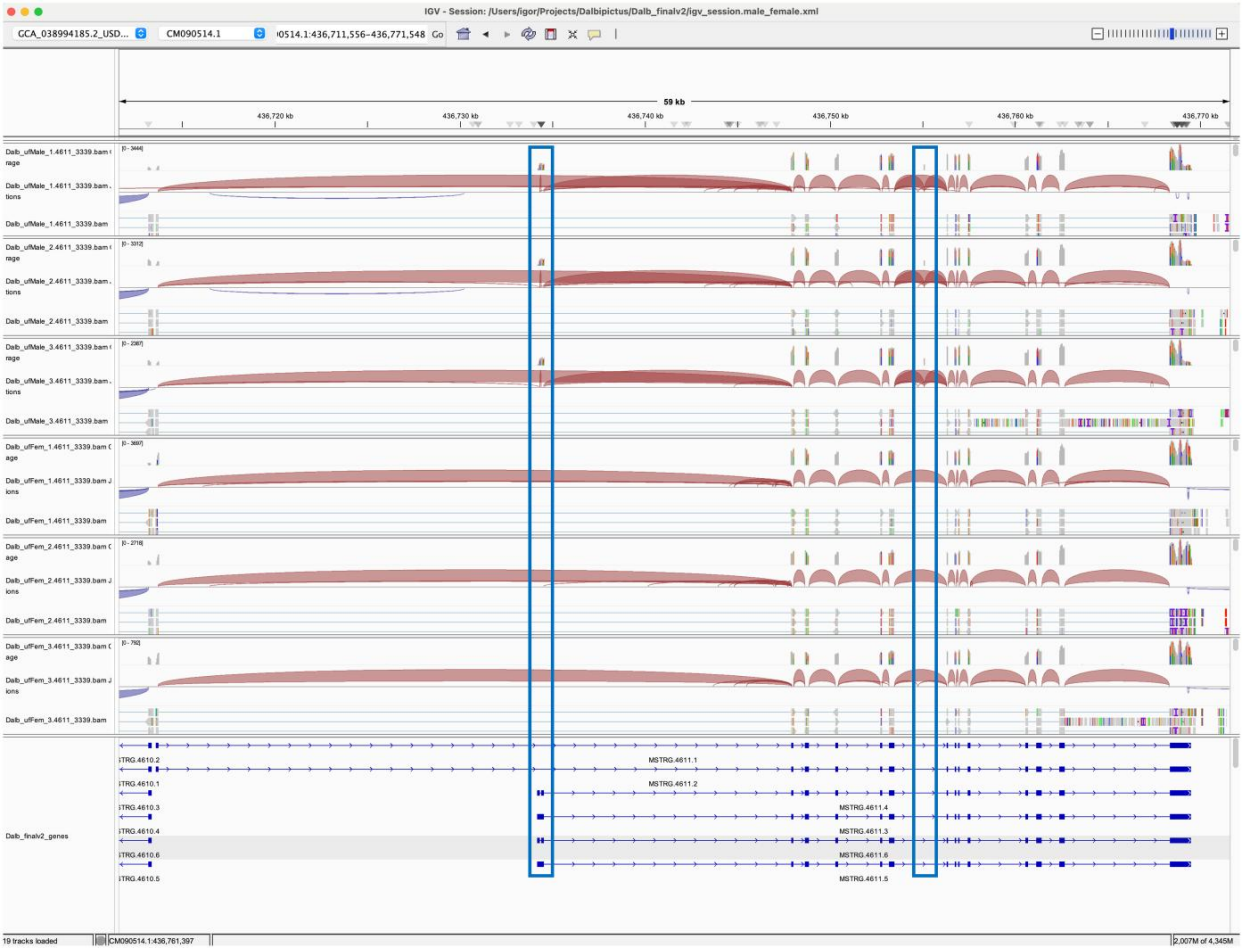
IGV transcript plot comparing male vs female *D. albipictus* splicing patterns for MSTRG.4611. Splice junctions are depicted as arcs connecting splice sites, and the arc thickness is proportional to spliced read counts for the junction. Rows 1–3 correspond to male samples and rows 4–6 to female samples. The bottom panel shows structures of 6 transcripts encoded by the MSTRG.4611 locus. Male-specific exon parts are highlighted with boxes. Exon parts 4, 5, and 6 representing alternative TSS are specific to transcripts MSTRG.4611.2-6; exon part 12 inserting the MLPV sequence into the active site is specific to MSTRG.4611.2-4.

**Fig. 6.**
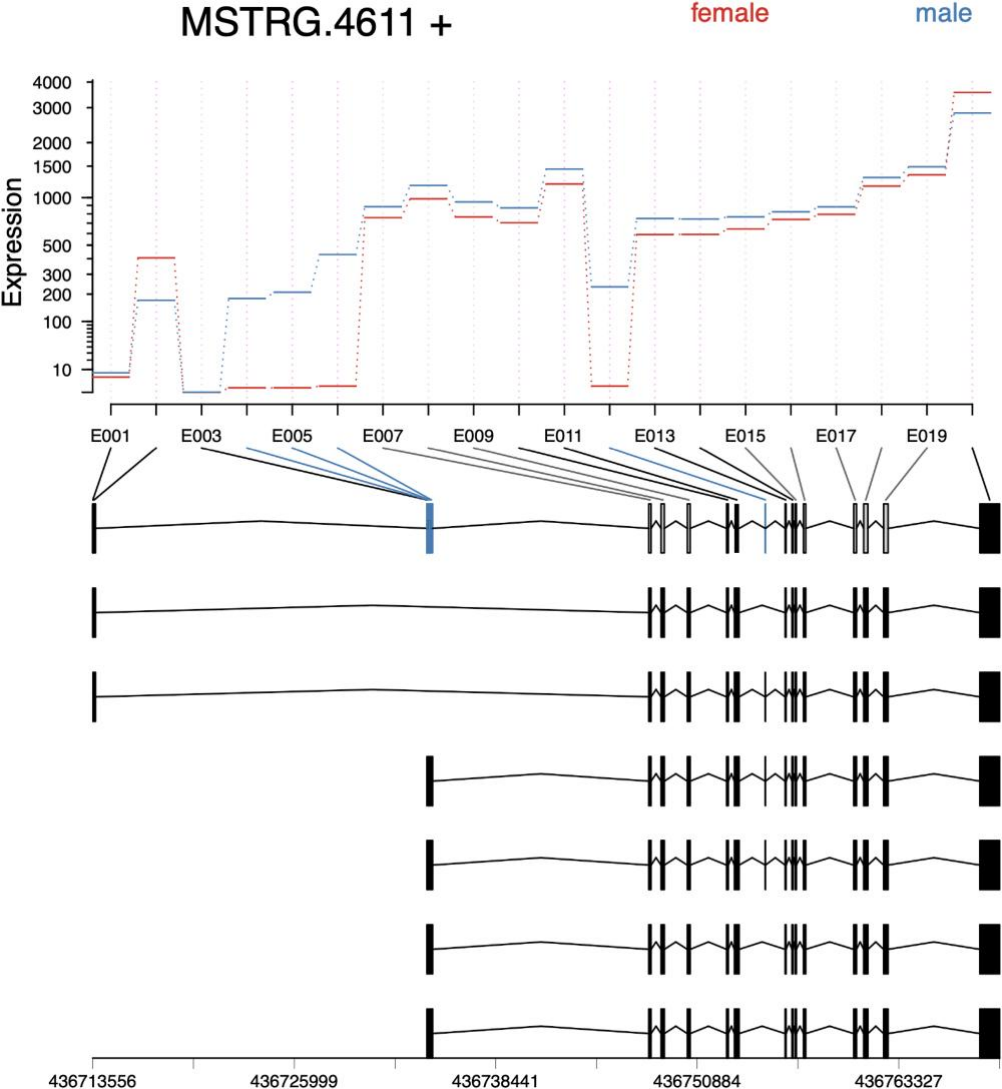
Exon plots analysis for MSTRG 4611 to elucidate sex specificity in *D. albipictus.* Exons were quantified, followed by differential usage analysis for the 3 ufMale compared to the 3 ufFem samples. Exons E004-6 and E012 show downregulation in female samples, suggesting these exons are male-specific.

For example, MSTRG.4611 encoded a homolog of a fatty acid CoA ligase Acsl3-like protein and had 6 annotated transcripts (Fig. 5). Exonic parts E004-6 and E012 were significantly downregulated in male samples (*P*adj < 1e-55; log2 fold change > 8) (Fig. 6). Splice junction counts confirmed male specificity of several junctions: SJ002 connected to the first 2 exons in isoforms MSTRG.4611.4 and MSTRG.4611.6, while SJ003 was specific to MSTRG.4611.3-6. Additionally, SJ008 and SJ010 spliced exonic part E012, which was present in transcripts MSTRG.46112.2-4 (Table 3). These findings indicate that 4 transcripts with transcription start sites (TSS) near CM090514.1:436734180 were male-specific, while 2 transcripts with TSS near CM090514.1:436713550 were expressed in both sexes. Protein alignment revealed that the male-specific exon E012 inserted an MLPV amino acid sequence into the active site of the eukaryotic long-chain fatty acid CoA synthetase domain (LC-FACS). This modification may alter the substrate specificity of male isoforms (Supplementary Fig. 1).

**Table 3.** Splice junction counts for *D. albipictus* MSTRG 4611 showing sex specificity of SJ002, SJ003, SJ008, and SJ010 (male), MSTRG 10562 showing sex specificity of SJ006 (male) and SJ005 and SJ007 (female enriched), and MSTRG 3339 showing sex specificity of SJ006, SJ009, and SJ010 (male).

Junction ID	ufFem_1	ufFem_2	ufFem_3	ufMale_1	ufMale_2	ufMale_3
MSTRG 4611						
MSTRG.4611:SJ001	1162	710	225	380	389	262
MSTRG.4611:SJ002	0	0	0	64	64	47
MSTRG.4611:SJ003	0	1	0	617	646	489
MSTRG.4611:SJ004	1544	920	229	1465	1479	1149
MSTRG.4611:SJ005	1036	728	224	1079	1219	874
MSTRG.4611:SJ006	1153	818	213	1266	1285	1022
MSTRG.4611:SJ007	1236	774	234	1171	1256	955
MSTRG.4611:SJ008	0	0	0	529	486	471
MSTRG.4611:SJ009	1199	855	251	741	875	593
MSTRG.4611:SJ010	0	0	0	551	484	467
MSTRG.4611:SJ011	1213	774	228	1276	1387	1001
MSTRG.4611:SJ012	1230	754	246	1202	1337	1001
MSTRG.4611:SJ013	1294	850	251	1207	1316	1061
MSTRG.4611:SJ014	1031	662	171	971	1013	768
MSTRG.4611:SJ015	1232	856	238	1164	1213	895
MSTRG.4611:SJ016	1297	947	278	1237	1300	1045
MSTRG.4611:SJ017	1506	1083	304	1399	1422	1129
MSTRG 10562						
MSTRG.10562:SJ001	155	114	140	1025	1048	814
MSTRG.10562:SJ002	124	99	116	835	905	714
MSTRG.10562:SJ003	102	88	97	766	759	635
MSTRG.10562:SJ004	128	110	129	996	1023	795
MSTRG.10562:SJ005	111	108	111	69	52	76
MSTRG.10562:SJ006	4	2	4	851	868	628
MSTRG.10562:SJ007	112	103	141	78	90	85
MSTRG.10562:SJ008	85	86	90	889	1092	870
MSTRG.10562:SJ009	93	89	83	1041	1087	906
MSTRG.10562:SJ010	100	88	92	898	1015	773
MSTRG.10562:SJ011	113	82	107	991	1093	881
MSTRG.10562:SJ012	107	84	76	870	1029	744
MSTRG.10562:SJ013	89	91	98	1000	1186	772
MSTRG.10562:SJ014	108	94	93	1010	1090	831
MSTRG.10562:SJ015	39	42	32	451	494	380
MSTRG.10562:SJ016	0	0	0	15	11	13
MSTRG 3339						
MSTRG.3339:SJ001	195	187	240	1176	1075	915
MSTRG.3339:SJ002	176	215	258	1213	991	908
MSTRG.3339:SJ003	157	135	190	922	916	697
MSTRG.3339:SJ004	181	237	242	1145	1171	909
MSTRG.3339:SJ005	187	196	211	1131	1018	890
MSTRG.3339:SJ006	0	2	0	106	147	83
MSTRG.3339:SJ007	28	24	46	899	737	637
MSTRG.3339:SJ008	132	111	168	124	162	117
MSTRG.3339:SJ009	0	0	0	31	27	29
MSTRG.3339:SJ010	0	0	0	95	116	65

**Fig. 7.**
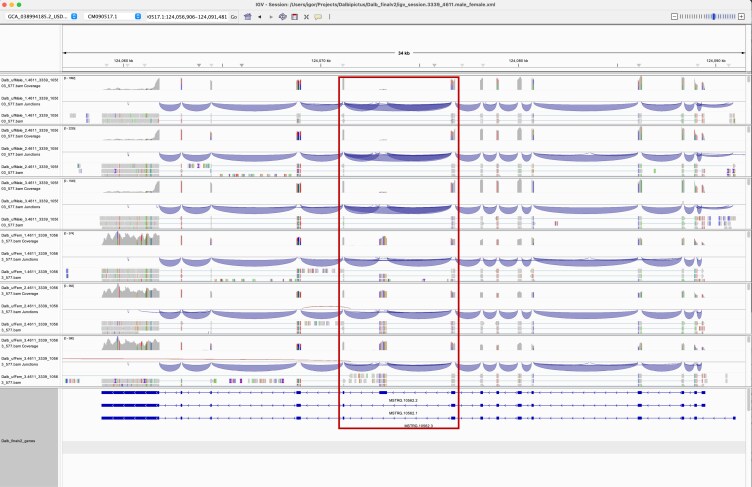
IGV transcript plot comparing male vs female *D. albipictus* splicing patterns for MSTRG.10562. Splice junctions are depicted as arcs connecting splice sites, and the arc thickness is proportional to spliced read counts for the junction. Rows 1–3 correspond to male samples and rows 4–6 to female samples. The bottom panel shows structures of 3 transcripts encoded by the MSTRG.10562 locus. Male-specific transcripts MSTRG.10562.1 and MSTRG.10562.3 lack exon part 7 deleting a 125 aa stretch in corresponding proteins. The location of the male-specific splice junction 6 is highlighted with a box.

**Fig. 8.**
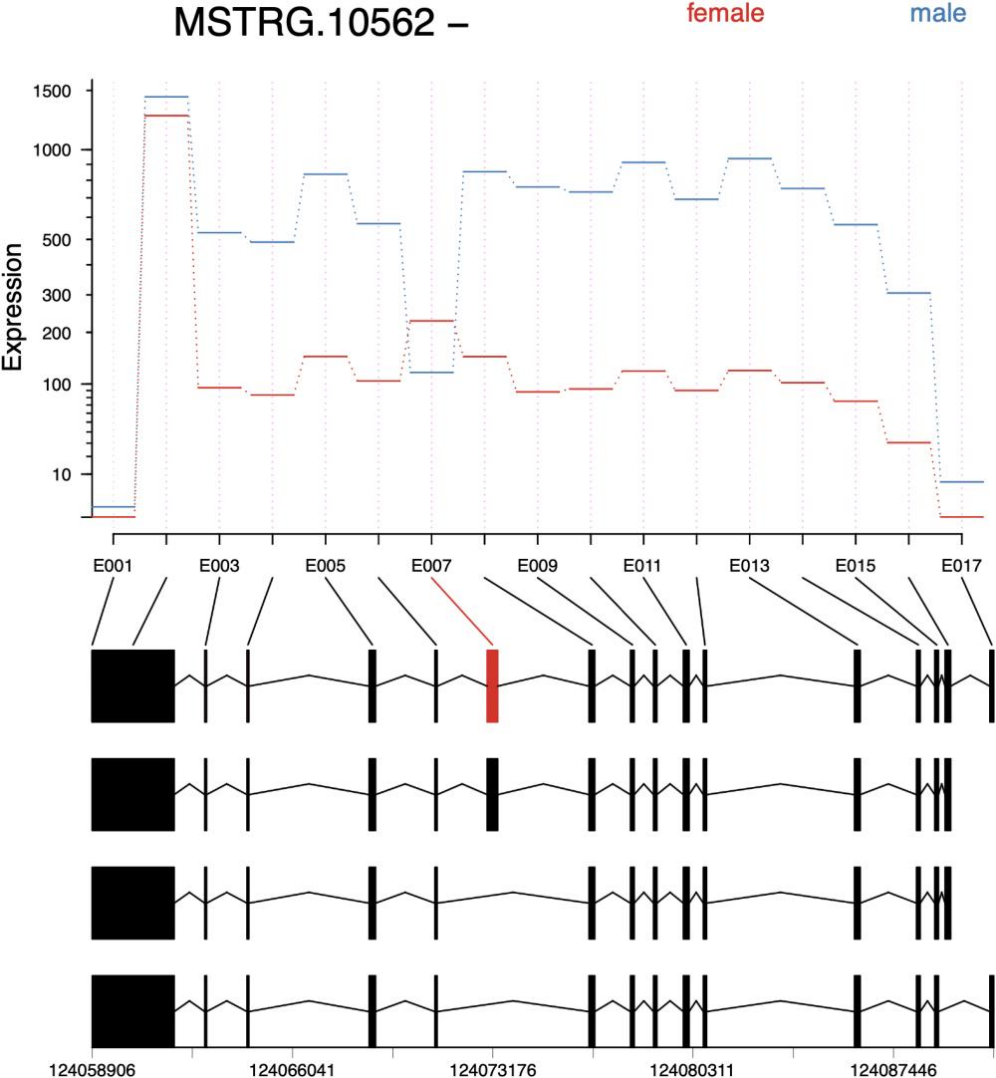
Exon plots analysis for MSTRG 10562 to elucidate sex specificity in *D. albipictus.* Exons were quantified, followed by differential usage analysis for the 3 ufMale compared to the 3 ufFem samples. Exons E007 show downregulation in male samples, suggesting this exon is female-specific.

Additionally, MSTRG.10562 encoded ralA-binding protein 1-like involved in small GTPase-mediated signal transduction and was composed of 3 transcripts (Fig. 7). Exon 7 was significantly downregulated in males (*P*adj = 2.4e-145; log2 fold change = 2.91) (Fig. 8). Splice junction counts showed that SJ006, which connected exons 6 and 8, was male-specific (Table 3). SJ005 and SJ007 were enriched in females. Together, these results confirmed that isoforms MSTRG.10562.1 and MSTRG.10562.3 were predominantly expressed in males (Figs. 7 and 8). Protein alignment indicated that male-specific isoforms lacked a 125-amino acid stretch in the second half of the protein (Supplementary Fig. 1). Although no conserved domains were identified in this region, it may play a role in modulating the efficiency of the GTPase activation cascade.

**Fig. 9.**
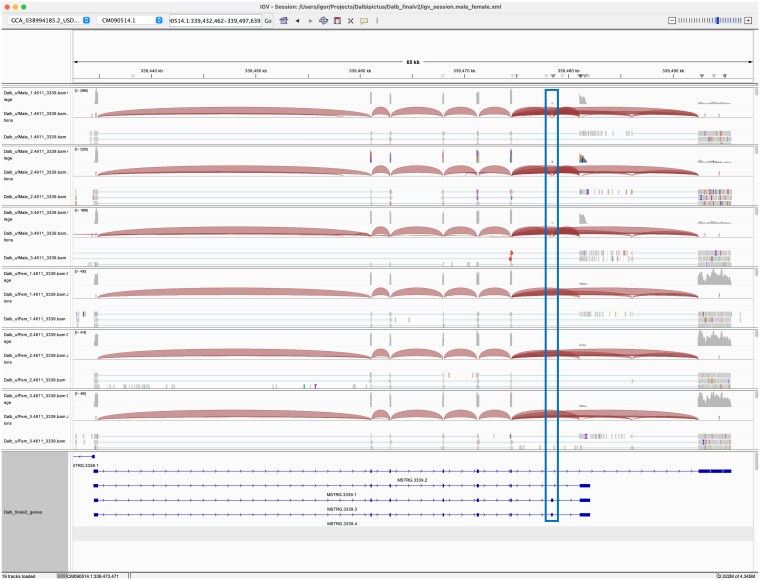
IGV transcript plot comparing male vs female *D. albipictus* splicing patterns for MSTRG.3339. Splice junctions are depicted as arcs connecting splice sites, and the arc thickness is proportional to spliced read counts for the junction. Rows 1–3 correspond to male samples and rows 4–6 to female samples. The bottom panel shows structures of 4 transcripts encoded by the MSTRG.3339 locus. Male-specific exon parts 9 and 10 present in isoforms MSTRG.3339.3 and MSTRG.3339.4 are highlighted with a box.

**Fig. 10.**
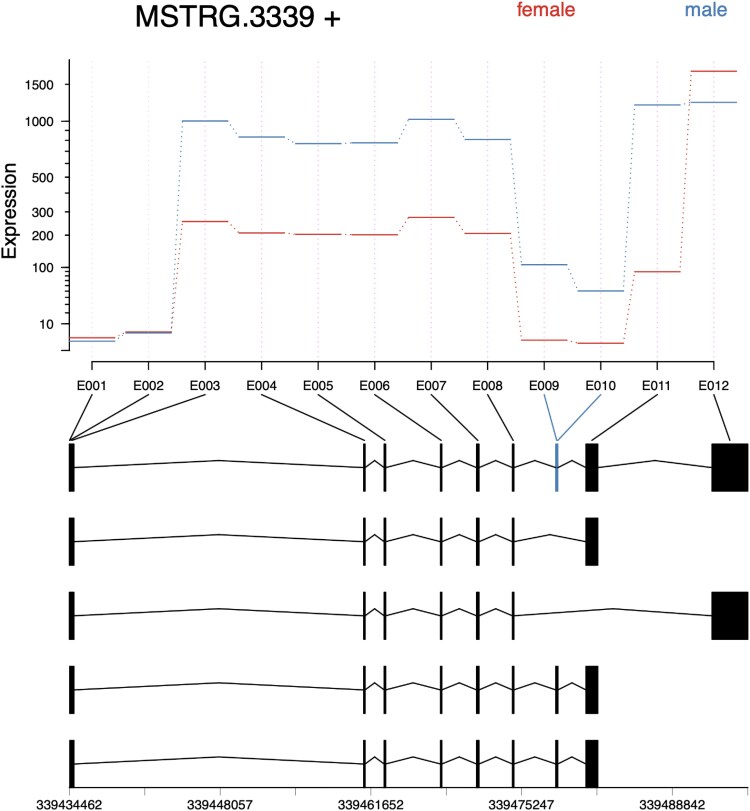
Exon plots analysis for MSTRG 3339 to elucidate sex specificity in *D. albipictus.* Exons were quantified, followed by differential usage analysis for the 3 ufMale compared to the 3 ufFem samples. Exons E009 and E010 show exclusive expression in male samples.

Lastly, MSTRG.3339 encoded a homolog of aquaporin-9 and expresses 4 isoforms (Fig. 9). Exonic parts E009 and E010—along with corresponding splice junctions SJ006, SJ009, and SJ010—were exclusively expressed in male samples and were associated with isoforms MSTRG.3339.3 and MSTRG.3339.4 (Fig. 10 and Table 3). Alternative splicing altered the carboxyl terminus of the encoded proteins, which lay just outside the major intrinsic protein domain predicted to form a membrane channel (Supplementary Fig. 1).

## Discussion

In this study, we generated high-quality transcriptome data for 5 *D. albipictus* sample types and used them to annotate the recently published *D. albipictus* genome. More than 79.95 million reads were collected per sample, with over 90% of reads successfully mapped and more than 85% uniquely mapped. These efforts resulted in the identification of 47,811 transcripts and 22,680 genes, of which 47.88% (10,860 genes) were annotated with GO functions and 58.39% (13,243 genes) with PFAM IDs. Significant homology was seen with *D. andersoni* and *D. silvarum*, 2 closely related tick species.

Differential gene expression analysis revealed significant variation in the activity of BPs across the 5 sample types. First, embryos showed upregulation of genes involved in DNA repair, replication, and transcription regulation compared to adult ticks. This reflects the high mitotic activity and significant developmental needs of embryos (Adryan and Teichmann 2010). Additionally, Mrs and Ov-preov both showed increased chromosomal condensation compared to their unfed adult counterparts, likely due to increased meiotic activity during spermatogenesis and oogenesis (Sonenshine *et al*. 2011; Wang *et al*. 2021; Kiyozumi and Ikawa 2022).

Compared to their respective reproductive tissues, the transcriptomes of unfed adult ticks were enriched with genes associated with facilitating successful blood feeding. For example, unfed female ticks showed overexpression of evasins, which modulate host immune responses by binding to chemokines and preventing leukocyte recruitment and maintain blood flow during feeding (Kazimírová and Štibrániová 2013). Unfed female ticks also showed overexpression of genes responsible for blood coagulation inhibitors (e.g. MSTRG.6585, MSTRG.6479), which promote blood feeding by reducing the risk of clotting (Kazimírová and Štibrániová 2013). Additionally, both unfed male and female ticks exhibited highly enriched GO:0030682 (symbiont-mediated perturbation of host defenses). This demonstrates that adult ticks are primed to evade or suppress the host immune defenses that are precipitated by blood feeding. Using genetic technology to knock out these enriched genes could potentially decrease blood feeding success and the transmission of tick-borne diseases.

A major focus of this study was identifying genes involved in sex determination pathways in order to potentially leverage them in sex-sorted genetic engineering. With this in mind, we identified 4 probable *dsx* homologs in *D. albipictus.* These are yet to be experimentally validated. Notably, MSTRG.10335 was highly expressed in both males and females but displayed significant splicing pattern differences; the precise splicing mechanisms were not identified. Sex-specific splicing of *dsx* is central to sexual differentiation in insects like *Drosophila melanogaster* (Burtis and Baker 1989), *Aedes aegypti* (Salvemini et al. 2011), and *Anopheles gambiae* (Scali et al. 2005). It has also been seen in crustaceans, albeit less frequently (Viet et al. 2022). To our knowledge, this is the first instance of sex-specific splicing of putative *dsx* in chelicerates. We suspect that this is, in part, because chelicerates have not been studied as robustly as insects and crustaceans in the past. We recommend that future studies analyze the transcription of *dsx* in other arthropods—particularly chelicerates—in an effort to better elucidate whether alternative splicing of *dsx* is a derived or ancestral trait.

The few studies that have investigated *dsx* expression in chelicerates have observed sex-biased expression (Pomerantz and Hoy 2015; Pomerantz et al. 2015; Gruzin et al. 2020). In this study, too, MSTRG.10337 was practically absent in female samples. The male-biased expression of MSTRG.10337 makes it a better candidate for genetic modification than MSTRG.10335 because it has more predictable and well-characterized promoter elements and lacks the complex posttranscriptional splicing machinery seen in sex-specific splicing processes. In particular, the simplicity of MSTRG.10337 can potentially be leveraged in pgSIT.

pgSIT is a powerful genetic tool that uses CRISPR-Cas9 technology to specifically disrupt genes that are essential for sex determination and fertility in an effort to reduce the population of arthropod vectors of disease (Raban, Gendron, and Akbari 2022; Raban *et al*. 2023; Weng, Masri, and Akbari 2024). It has been successfully developed for multiple insect species, for example, *D. melanogaster* (Kandul *et al*. 2019; Kandul, Liu, and Akbari 2021), *Drosophila suzukii* (Kandul *et al*. 2022 ; Witherbee and Gamez 2025), *A. aegypti* (Li *et al*. 2024), and *A. gambiae* (Apte *et al*. 2024). Similarly, CRISPR-Cas9 technologies have also targeted knockouts of female-specific isoforms of *dsx* in *A. gambiae* and have resulted in intersex individuals incapable of blood feeding or reproducing (Kyrou *et al*. 2018). These could be developed to target and knock out the MSTRG.10335 gene in *D. albipictus*, thereby creating sterile or intersex males. These genetically modified males could be released into the wild and compete with wild-type males for mates. Their successful mating would result in a reduction of the *D. albipictus* population and a subsequent reduction of tick-borne diseases.

In summary, the transcriptomic results of this study present an advancement in understanding the molecular mechanisms underlying biologic processes and the variation of biological activity across different life stages and tissue types in winter ticks. This information can be used to create genetic tools that provide effective population control and reduce the risk of tick-borne illnesses on wildlife, livestock, and public health.

## Data Availability

The Illumina sequencing reads for the *D. albipictus* embryos, male reproductive system, ovaries preoviposition, unfed males, and unfed females have been submitted to the NCBI Sequence Read Archive within the Transcriptome database (BioProject Accession Primary PRJNA1072088; RNA-Seq SRA study SRP492691, SRR28628997, SRR28628998, SRR28628999, SRR28629000, and SRR28629001). Supplemental Material available is at figshare: https://doi.org/10.25387/g3.28920842.
